# FEASIBILITY OF AN INTERACTIVE, ITERATIVE ZOOM-BASED ANTI-RACISM COURSE FOR ACADEMIC NEUROLOGY FACULTY

**DOI:** 10.21203/rs.3.rs-7087000/v1

**Published:** 2025-08-25

**Authors:** Neishay Ayub, Shreya Vakharia, Joshua Ray Tanzer, Ryan Lantini, Rochelle K Rosen, Debasree Banerjee

**Affiliations:** 1 Department of Neurology, Brown University; Providence, United States; 2 Department of Medicine, Brown University; Providence, United States; 3 Brown University Health Biostatistics Epidemiology Research Design Informatics Core; Providence, United States; 4 Center for Behavioral and Preventive Medicine, The Miriam Hospital, Brown University, Providence, United States

**Keywords:** antiracist-racism, equity, neurology, medical education

## Abstract

**Background:**

While there has been increasing education on diversity, equity, and inclusion (DEI) for medical students and residents, neurology faculty are also in need of formal education on race and racism. The aim was to implement and evaluate the feasibility, efficacy and preliminary impact of an interactive Zoom-based anti-racism curriculum that was repeated in a new academic year to foster learning and discussion amongst neurology faculty.

**Methods:**

A Justice Equity Diversity and Inclusion Curriculum (JEDI) was delivered to Brown University Neurology faculty during 2021–2022 and again in 2023–2024. The curriculum consisted of seven Zoom-based didactics sessions and group discussions led by institutional experts in DEI. A pre- and post-curriculum survey was sent to participants, assessing attitudes, interest, and knowledge about race and racism in medicine, confidence and comfort in addressing racism, curricular and departmental assessment, and feedback.

**Results:**

Most Brown Neurology faculty (n=36, 90%) participated in at least one session of the first round of implementation. Almost all faculty members endorsed the utility of an anti-racism education, and most reported continued interest. Participants reported increased concern regarding the use of race in medical algorithms as well as increased confidence in race-based discussions. This effect was most prominent among faculty members with less than ten years of experience. Qualitative data illustrated the faculty’s awareness of racism in neurology as well as a need for continued anti-racism education. Repeating the curriculum was viewed favorably.

**Conclusions:**

The majority of academic neurology faculty at our institution recognize a need for an anti-racism curriculum. Interactive, zoom-based sessions are a feasible medium for education and fostering dialogue about race and racism. Repeating anti-racism education is helpful for medical academicians.

## Background:

Systemic racism negatively impacts neurologic health outcomes and contributes to health disparities.^1^ Black and LatinX individuals with neurologic diseases are less likely to see an outpatient neurologist compared to White individuals^1^ and are also more likely to be treated in an emergency department, have more hospital stays and have higher per capita inpatient expenditures.^1^ Subspecialty care is also disparate with Black individuals being less likely to be evaluated by an epileptologist or undergo epilepsy surgery compared to White individuals.^2^ Similarly, there are health inequities for neurologic emergencies such that Black individuals are less likely to receive tissue plasminogen activator or mechanical thrombectomy and are more likely to have worse stroke outcomes relative to White individuals.^3–4^

Considering these health disparities, there has been an increased dedication to anti-racism curriculum and evaluation of systemic racism at the medical school, neurology residency and neurology national society levels.^5–7^ There has been an introduction of institutional diversity, equity and inclusion (DEI) education for neurology residents, as seen at academic centers throughout the country.^5,8^ While training the next generation of physicians is essential, there is also an urgent need to train current faculty to become effective anti-racist preceptors and clinicians.^9^ Until recently, most DEI education has been cultural sensitivity training and implicit bias training, which improve foundational knowledge but are distinct from an anti-racist curriculum, which provides the framework to recognize and react to *racism* in medicine. The format of these types of training also does not often allow for deep discussions.^9–11^ To address this need, we implemented an interactive Zoom-based anti-racism curriculum for the neurology faculty.

Our curriculum was based on interpretive theory to increase the perceived significance of racism in medicine,^12^ specifically to improve awareness and foster discussion about racism among Neurology Faculty via an interactive longitudinal anti-racism curriculum in medical practice (patient-facing roles and as academic scholars and educators). The materials were created to touch upon several andragogical cognitive and affective learning goals such as “caring,” “human dimension,” “foundation knowledge”, “application,” and “integration”. This curriculum was successfully employed in a medicine division pilot study.^13^ This curriculum was tailored to the neurology department and the iteration was revised based on feedback from the first cycle.

## Methods & Curriculum Description:

### Participants:

All neurology faculty at Brown University were invited via email, to participate in the research portion of the intervention in the academic year 2021–2022 (n=36) and write-in feedback for the second curriculum cycle was requested in 2024 (n=43). The education was available for all faculty to attend. Informed consent was waived by the Lifespan Institutional Review Board (#1634342).

### Curriculum Description.

The curriculum was developed by the Justice Equity Diversity Inclusion (JEDI) Committee at Brown Physicians Incorporated, focusing on a growth mindset.^13^ There were seven 1-hour interactive Zoom-based sessions. The course was developed in an andragogical framework, using published scholarly work in DEI and content by Brown Advocates for Social Change and Equity, tailored for a faculty audience, revised by Black Indigenous People of Color (BIPOC) and White allies who are DEI experts in medicine. The first four sessions provided anti-racist racism education, and the last three sessions focused on tools for dismantling racism (Table 1). Each session included an experiential and participatory component through exercises and guided discussions that fostered self-reflection and promoted an inclusive environment. Sessions were led by trained clinicians outside of the neurology department to decenter hierarchy within the neurology department. A more complete description of the modules can be found in the appendix (appendix 1). Between the first cycle and its second iteration, content and material were continually updated, but the general topics were consistent.

### Curriculum Assessment:

A mixed quantitative and qualitative pre- and post-assessment was performed (appendix 2) for the 2022 series via a secure online research database REDCap^14^ that included six questionnaires on the following topics: 1) general beliefs about race-based discrimination in healthcare, 2) awareness of the use of race in medical algorithms, 3) definitions of terms related to racial bias, 4) personal comfort and confidence with talking about racism, 5) department level assessment of commitment to equity, and 6) personal commitment to taking action to inform oneself about bias in medicine.^15^ Those who completed a pre-survey received a post-survey personalized link. Public post-survey links were also disseminated to capture faculty who did not attend the first session, and the data reported is a pooled sample of linked and unlinked surveys.

Open-ended survey Questions were included to assess: 1) awareness of racism and its consequences for patients, staff, and healthcare providers; 2) curricular feedback; 3) perception of racial equity personally and professionally within their department; and 4) the impact of the curriculum in their personal and professional lives. In 2024, after the second delivery of JEDI, participants were invited to participate in a voluntary post-intervention survey (public QR code displayed during the last session, with email follow-up) to assess prior participation and impressions about the utility/deficiencies of the current iteration.

### Statistical Analysis:

All statistical analysis was conducted using SAS software. Counts (percentages) of examined variables are presented. A validity analysis was performed with multi-level modeling, modeling the average scores on the six questionnaires nested within time points nested within individuals. A factor analysis model was used to understand the multivariate relationships between the questionnaires. This approach allowed us to estimate the relationships between the questionnaires jointly and the extent to which the average scores changed across the questionnaires before and after the curriculum. A Bayesian model was implemented to mitigate sampling bias due to missingness and nonresponse. A detailed statistical analysis is available in the appendix (appendix 3).

### Qualitative Data:

Open-ended survey responses were entered into NVivo 20 software to aid qualitative content analysis by authors (RL, RKR).^16^ An analytical code book containing deductive and inductive codes was created. Deductive codes represented the open-ended questions with affirmative or negative answers (for example, “Do you believe there is discrimination against clinicians?”, “Do you believe patients are treated differently based on their race?” and “What would you change about the curriculum?”). Inductive codes were created to identify themes raised by the participants within those open-ended responses, such as the specific types of bias identified and specific recommended changes. Summaries were written for all relevant codes and synthesized for themes by authors (NA) and qualitative experts (RL, RKR)

## Results & Assessment Data:

### Demographics:

Table 2 provides participant characteristics and faculty attendance for the first 2021–2022 cycle. Most faculty participated in at least one session (n=36, 90%). Faculty respondents consisted of 57% junior faculty (n=17) (within their first ten years of practice) and 44% senior faculty (n=12). Six (15%) participants completed both pre- and post-intervention surveys. In comparison, 24 (60%) participants completed pre-intervention surveys only and 15 (37.5%) participants only completed post-intervention surveys (nine of those respondents used an unlinked post-survey link).

### Quantitative Analyses:

We found that participants reported a greater understanding of the problems of bias in medicine after completing the curriculum, particularly among more junior faculty members (Table 4). Junior faculty members demonstrated meaningful increases across questionnaire domains (${\hat{\beta }}_{\Delta }=0.62$, 95% CI [0.26, 0.95]), which was not seen for senior faculty members (${\hat{\beta }}_{\Delta }=0.26$, 95% CI [−0.31, 0.84]). A validity analysis was performed, which can be found in the appendix. Participants who reported being most comfortable talking about race ($\hat{\lambda }=0.73$, 95% CI[0.39, 1.14]) and those who most strongly reported that their department was committed to equity ($\hat{\lambda }=0.81$, 95% CI[0.47, 1.15]), reported less concern about bias in medicine ($\hat{\lambda }=-0.49$, 95% CI[−0.85, −0.11]) and were less likely to accurately define forms of bias ($\hat{\lambda }=-0.37$, 95% CI[−0.71, −0.02]).

### Beliefs:

At the individual level, there was already a high baseline belief that racism exists in medicine and that it has significant effects on both patients and the healthcare team. Before the intervention, 92% (n=22) of faculty survey participants wanted an anti-racism curriculum and 96% (n=23) of faculty respondents believed that race-based discrimination exists and were concerned about its consequences. After the curriculum, all respondents (n= 13, 100%) believed that race-based discrimination occurs. Both before and after the curricula, most faculty felt that racism occurs against healthcare providers, staff, and patients with significant consequences for patient care.

### Algorithms/Definitions:

Respondents had varying awareness of how race is used in medical algorithms. Before the curriculum, faculty correctly identified whether medical algorithms included race anywhere from 4–79% (Mean (M) = 25% correct); after the curriculum, the range increased from 0–92% (M = 48% correct). There was a slight improvement in the ability to define microaggressions, race, bias, and diversity terms from 54–100% (M = 86% correct), prior to the curriculum to 63–100% (M = 89% correct) after the curriculum (Table 3).

### Comfort:

Comparing pre- and post-curricular responses, more faculty agreed that they could identify examples of institutional 58%, (n=14) vs. 100%, (n=11), interpersonal 79%, (n=19) vs 100%, (n=11) and structural racism 71%, (n= 17) vs 91%, (n=10). There was moderate comfort in initiating conversations about race and being comfortable when others discussed race before the intervention, 42%, (n=10) and 67%, (n= 16). After the curriculum, however, all faculty participants reported comfort with discussing race and being around others discussing race. Participants reported increased beliefs that they had the resources or tools to achieve racial equity after the curriculum (33%, (n=8) vs 72%, (n=8)).

### Departmental Perception:

There was increased faculty perception of the departmental commitment to racial equity and providing equal opportunity after the intervention (7.5%, (n=21) and 67%, (n=16); vs 100%, (n=11) and 90%, (n=10)) respectively.

### Personal Action:

Faculty participants reported personal action in advancing racial equity in daily clinical practice at 75%, (n=18) before and 91% (n=10) after the curriculum. Faculty participants reported similar efforts to educate themselves about the experience of Black, Indigenous and Persons of Color and how to improve racial equity before and after the intervention.

## Qualitative Analysis:

Qualitative data from the 2022 cycle was analyzed under three topics: participants’ awareness of race and racism in medicine, their department-level view of racial equity, and their assessment of the curriculum. Illustrative quotations are shown in Table 5.

### Awareness of race and racism in medicine:

Several participants (n= 4) note that racial discrimination of patients impacts healthcare access and delivery. One participant links racism to health outcomes: “*There are several studies supporting the fact that there are worse health outcomes for minorities”* [participant 4]. There was also, however, some ambivalence expressed as to the degree that racism exists in medicine. Specifically, two participants expressed concern over generalized statements about patients and healthcare providers being treated differently due to race. One reported having read articles about “*… how [B]lack MDs experience racist statements*” and then added that they have also witnessed White physicians on the receiving end of racist behavior. They suggest that this behavior is not necessarily related to race but to the “*culture of medicine*” [participant 5]. Another respondent expressed a similar concern suggesting, “*Sometimes patients are treated differently, sometimes they are not. It depends on the setting and situation, not a blanket statement”* [participant 6].

### Departmental-level view of racial equity:

Several faculty (n=12) recommended continued focus on an anti-racist racism curriculum. Recommendations by faculty to specifically continue opportunities for anti-racist racism(n=9). Examples include: “*encourag[ing] GR [grand rounds] speakers to include information on potential influences of discrimination and racism on health outcomes, when relevant to the topic*” [Participant 7] and “*…provid[ing] additional opportunities for education on promoting equity and recognizing racism in medicine…”* [Participant 7] with protected time for this work. Another respondent states a focus on equity could be achieved by: *“Continuing to provide safe spaces for education and discussion regarding race … and how institutions and individuals can improve appreciation and understanding of cultural differences”* [participant 9]. This respondent also comments on the need for mentorship to improve the pipeline of racially diverse neurologists: “*Work on inclusion earlier on (foster interest in neurology in high school etc., provide mentorship along the way to encourage interested students)*” [participant 9].

Some respondents (n=3) emphasize a greater focus on institutional levels of racism. One respondent calls for a *“…review of all policies, medical record templates, laboratory test result templates, periodic performance review templates, and hiring policies for examples of institutional racism”* [Participant 10]. Another respondent writes about patient and provider equity as influenced by the patterns of patient referrals: *“Often providers of color are given patients of color, which can burden those providers with navigating additional barriers that other patients/providers do not need to tackle”* [participant 11].

Many faculty (n=9) indicate a need to examine and restructure hiring practices. One writes that they hope to see increased diversity in faculty hires: “*continue[d] efforts to create greater diversity among faculty, clinicians, and those in support roles when filling vacant positions in the department*” [participant 7]. Others state these efforts should also be applied to faculty promotion and one emphasizes the importance of tracking promotion data.

Finally, three respondents referenced a focus on patient care and delivery. One writes that a departmental emphasis on equity would help by “*providing better access to patients without insurance or underinsured*” [participant 13].

### Curriculum Effectiveness Appraisal:

Participants had varied views on the efficacy of the JEDI Curriculum. Several elaborated on the usefulness of the curricula as a whole. For example, one respondent notes, “…*this [JEDI curriculum] is a step in the right direction*” [participant 14]. Another states that JEDI should continue as a “*…lecture series… available on an ongoing basis*” [participant 4]. One felt the curriculum helped them as a healthcare provider because it allowed them “*to recognize situations*” [Participant 15]. Another writes explicitly about a change in their perception of treating race in medical education: “*Prior to the course, my interaction with patients, students, etc. has been ‘color-blind’*. Another participant wrote, “*… [the session] was illuminating due to the understanding of what constitutes ‘diversity’ in the educational realm*” [participant 16].

One respondent expressed concern with the curriculum. They commend the attempt to identify the issue of racism in medicine but also state that the topics of the curriculum are problematic because they are based on “*Critical Race Theory”*. They believe that there should be “*more evidence-based studies in medicine*” about the topic and “*less exercises in social re-engineering proselytizing on Western, white oppression over the centuries.”* [participant 6]

There was variation regarding the sessions that were described as most or least helpful. Two participants identified the session on genetics and race as biology the least helpful in one case because they were having “…*trouble understanding the genetics”* [participant 8]. The power and privilege session was divisive, particularly the flower power exercise. Two participants indicated they liked the ‘Flower Power’ session, with one saying, “*I had never examined my potential biases or privileges like that*. …*For those of us that are visual learners, it was very illustrating*” [participant 4]. In contrast, another participant thought the flower power exercise was the least helpful because the “… *solution to addressing racism is not being sorry for one’s race or ‘lack of diversity’” [participant 6]*. One participant considered the historical sessions to be the least beneficial: “*Honestly, all of the ones that I attended were helpful, instructive, and illuminating*… [the historical sessions] *were a little dry, which can lead to tuning out*” [participant 4]. One participant could only attend one session but did say that **the** session on training and equity “…*was illuminating due to the understanding of what constitutes ‘diversity’ in the educational realm” [participant 16]*. One participant noted *the training on being mindful of microaggression was very helpful!”* [participant 17]. Finally, one participant thought the format of the sessions was great, and another found the breakout sessions to be the most useful.

## 2024 cycle:

Few participants completed the post-intervention survey for the second cycle (n=12). All responders had participated in the inaugural cycle and on average, participants endorsed using the skills they learned during the first JEDI cycle 3.4 on a 5-point Likert scale (3 = sometimes, 4= often). See Table 6 for the strengths and weaknesses reported by faculty of the 2024 JEDI curriculum. Topics respondents hoped to see for future JEDI sessions included race and health access, expanding the pool of diverse providers, and how to promote inclusivity with trainees among other topics.

## Discussion:

While several studies have evaluated diversity curricula for neurology residents, this is one of the first to assess the feasibility and efficacy of an anti-racism curriculum for neurology faculty.^5,7–8,17^ It is encouraging that despite high baseline levels of awareness of racism and bias and their effects on patients and the medical team, we demonstrate improvement in belief, medical knowledge and commitment to action by participants. We observed disjoint relationships between perceiving and understanding bias among participants, a known phenomenon. This speaks to a Dunning-Kruger effect, likely a barrier to a growth mindset.^18–19^ Similarly, an intriguing variance around the degree of change between junior and senior faculty may result from more recent advancements to include anti-racist racism in medical school^20–21^ and graduate medical education. The curriculum did not improve medical knowledge of the biologization of race in medical algorithms, possibly due to the algorithms tested not being neurology specific.

Current faculty need focused, intentional curricula to explore their understanding of diversity and bias to educate future physicians, particularly regarding several policies that threaten to widen the gap in healthcare for minoritized groups.^9^ We focused on gaining specific skills: 1) learning vocabulary to discuss races and racism (as seen with improved scores on definitions), 2) increasing confidence in knowledge about racism (as evidenced by improved self-reported recognition of acts of racism and improved scores on having the tools and resources to combat racism), 3) and having practiced discussions during the curriculum (which is demonstrated in qualitative data acknowledging how the sessions provided a safe space for discussion). This anti-racism curriculum helped participants have a greater ability to engage in difficult conversations about these sensitive topics. While there was no noted change in self-education about race and racism among faculty, there was improved self-reported action to promote equity in daily clinical practice. Baseline interest in this topic is likely due to self-motivated engagement as reported on the survey and efforts by the department. In our qualitative data, faculty offered multiple specific examples of institutional racism that can serve as targets for policy change. This demonstrates that a longitudinal, interactive model can support adaptive behavior.

At the group level, there were also improvements in views about the department and its role in advancing equity after the intervention. This may reflect the presence of departmental leadership during the sessions and support for the session times (which replaced faculty meetings)^22^. The slight decline in the percentage of faculty who felt that the department provides equal opportunities to all may be due to greater awareness of inequities in the group after the completion of the curriculum.

A specific strength of this study is that it shows that the iteration of the anti-racism curriculum is well received and generally thought to be helpful for faculty, underpinning the nuance and challenge of internalizing this material for learners.^23^

There are several limitations to this study. This single-center intervention was conducted during the first round of the pandemic. Response rates for the pre-and post-survey were not robust, which may have affected interpretation due to selection bias. While the epistemological dimensions of anti-racist racism are difficult to capture, the pre-and post-intervention survey method did not robustly assess long-term follow-up data to assess *behavior* impact, however participants in the second cycle reported using skills they had learned during the first cycle supporting behavior change and skills development from this curriculum. Another limitation is respondent selection bias as attendance and survey completion were optional, with a decrease in survey response in the post-intervention collection. We used an analytic approach that seeks to mathematically address known problems with data collection, but this remains a limitation, and the interpretation of results must be considered accordingly.

The success of this curriculum lies in the andragogical approach, use of published studies as supporting data, outlined goals, multiple points of engagement with different audience sizes and formats and ways to participate as well as summary statements and takeaway points. Compared to other published curricula,^5,7–8,17, 24^ a key component was the focus on creating safe spaces by incorporating reflective observation for individual exercises and small and large group discussions. Additionally, after completing the first and second cycles, the department endorsed the utility of repeating the curriculum. This is particularly important in light of the scrutiny anti-racism education has faced since the pandemic^25^. It also makes the discussion surrounding how to disseminate knowledge around racism, an inclusive learning environment for willing participants of all sociopolitical beliefs both challenging and of utmost importance.

## Conclusion:

Implementation of an ongoing anti-racism curriculum can be effective in improving knowledge about racism in medicine, changing attitudes and increasing intention to change behavior to combat racism in an academic neurology department.

## Figures and Tables

**Table 1:** Session Topics. Session Description including Name, Objectives, Content and Exercise

Topic	Objectives	Content	Exercise
*Race and Its History*	Reflect on our own experience with race and racism Describe the origins of “race.” Identify examples of race and racism in our daily lives and as medical practitioners	Definition of race as a social construct Definition of systemic racism Origins of the racial categories and its (erroneous) roots in scientific theory (Carl Linnaeus, Systema Natura)	Self-reflection about when the participant first identified the concept of race and racism
*Myth of Race as Biology*	Describe historical and contemporary examples of race presumed to have biological origins Understand the evolution of skin pigmentation Discuss The human genome project Identify how it applies to us today, e.g., algorithms, census tracking	Historical examples of biologization of race (and its racist effects) Evolution of racial category for the national census Current theory of evolution of skin pigmentation Human genome project in the context of racial categories	Flipped classroom, faculty was given the Rosenberg et al, paper to review prior to the session. Large group discussions about the current use of race in clinical medical neurology practice. Small group discussion about how faculty teach about race and its importance in neurology. Large group discussion about the use of race in medical algorithms
*Racism in Medicine*	Define racism and describe racist policies and ideas Differentiate Implicit and Explicit Bias Describe the Levels of Racism Discuss Examples of Racism in Medicine Brainstorm leaks in the faculty pipeline	Definitions of implicit and explicit bias Levels of racism: internalized, personally mediated and institutionalized as outlined by A Gardener’s Tale.	Small group exercise about how levels of racism affect the neurology pipeline Large group Discussion of the individual/departmental role in breaking the cycle of racism.
*Power and Privilege*	Define power and privilege Reflect on our own identities Define White Privilege	Definitions of power and privilege and white privilege both economically and within medicine.	Flower power exercise Small and large group discussion of perspective: how one’s view of themselves compares to how society views them and how this factors into inequity in medicine. “A day in the life” exercise (for personal life and medical professional life)
*Interpersonal Racism*	Review examples of daily racism in medicine Define microaggressions and identify examples Define bystander effect Illustrate how to be an active bystander	Definition of microaggressions Upstander techniques	Practice exercises on naming microaggressions from Carnes et al, BRIM Volunteering participants’ lived experiences Role play: how to respond as a victim, bystander or perpetrator
*Balancing Training and Equity*	Review lack of equipoise in training and patient outcome Describe methods to improve diversity in Medical schools Discuss trainee data	Segregation of patient care in trainee versus faculty clinics Barriers to medical school, residency, and fellowship matriculation	Large group data analysis Neurology faculty evaluated internal data for residency applicants and matriculants Small group discussion on ways to improve diversity within trainee cohorts.
*Race in Research*	Appreciate the history of race in research Examples of racism in research Outline Pitfalls in using race in research Define our role as physician-scientists	Historical and current examples of racism in research How to use and interpret racial categories in research	Large group discussions focused on strategies better to address the sociopolitical nature of racial categories in research.

**Table 2:** 2021–2023 Participant Characteristics and Attendance. Survey responses for participant demographics and attendance. Descriptive Data is presented as a frequency (n) and percentage (%; where appropriate)

Participant Traits	Statistic				
	**1–5 vears**	**6–10 vears**	**11–20 vears**	**>20 vears**	
Years in practice (n, %)	15 (50%)	2 (7%)	8 (27%)	5 (17%)	
	**Inpatient**	**Outpatient**	**Both**		
Clinical practice (n, %)	2 (7%)	5 (15%)	26 (79%)		
	**Yes**	**No**	**Not Sure**		
Reported interest in antiracisanti-racismum pre training (n, %)*	22 (92%)	1 (4%)	1 (4%)		
	**Stronglv Agree**	**Agree**	**Neither Agree nor Disagree**	**Disagree**	**Stronglv Disagree**
Reported involvement in advancing racial equity in daily clinical practice pre-training (n, %)**	2 (8%)	14 (58%)	6 (25%)	2 (8%)	0 (0%)
**Participant Attendance**					
Number of sessions (M, SD)			4.29 (2.37)		
Completed at least one session (n, %)			36 (90%)		
Session: race in history (n, %)			10 (25%)		
Session: myth of race as biology (n, %)			6 (15%)		
Session: racism in medicine (n, %)			10 (25%)		
Session: power and privilege (n, %)			9 (22.5%)		
Session: dismantling structural racism (n, %)			11 (27.5%)		
Session: training vs equity (n, %)			8 (20%)		
Session: race in research (n, %)			6 (15%)		
Pre surveys completed			24		
Post surveys completed			15		
Total participants survey responses (Pre/Post)			40		

* This question was analyzed in the aggregate in the beliefs subsection of questions. This specific detail is reported because it was identified as a theme among qualitative findings.

** This question was originally included in the comfort subsection of questions, however it demonstrated greater relevance to the action subset of questions. It was not included in the aggregate analysis because the scaling was different (Likert scale from 1 – 5 as opposed to “Yes”, “No”, and “Not Sure”).

**Table 3:** Pre/Post Curricula Survey Responses for Six Subsections (Beliefs, Algorithms, Definitions, Comfort, Departmental, Action). Descriptive Data is presented as a frequency (n) and percentage (%). “Agree” represents combined responses for “Very Strongly Agree” and “Agree” while “Disagree

*Beliefs*: Question	Yes (Pre/Post) n (%)	No (Pre/Post) n (%)	Not Sure (Pre/Post) n (%)
1. Do you want a continued structured curriculum on anti-racism awareness available to you during your medical practice?	22(92)/11(85)	1(4)/2(15)	1(4)/0(0)
2. Do you believe discrimination based on race occurs in medicine?	23(96)/13 (100)	1(4)/0(0)	0(0)/0(0)
3. Are you concerned about the existence of discrimination in medicine?	23(96)/11(79)	1(4)/1(7)	0(0)/1(7)
4. Do you believe there is discrimination of physicians, PAs, and/or NPs based on race?	21(88)/12(92)	1(4)/1(8)	2(8)/0(0)
5. Do you believe there is discrimination against nurses, nursing assistants, physical therapists, occupational therapists, and respiratory therapists based on race?	21(88)/11(84)	1(4)/1(8)	2(8)/1(8)
6. Do you believe patients are treated differently based on their race?	21(95)/11(84)	0(0)/1(8)	1(5)/1(8)
7. If yes (to Q6), do you think it has significant consequences for patients?	20(100)/8(100)	0(0)/0(0)	0(0)/0(0)
*Algorithms*: Questions		Accurate N(%) Pre/Post	Inaccurate N(%) Pre/Post
Which of the following incorporate race in the score/algorithm (check all that apply)? eGFR		19 (79)/12 (92)	5(21)/1(8)
PFTs		7(29)/5(38)	17(71)/8(62)
National Cancer Institute: Breast Cancer Risk Assessment Tool		6(25)/5 (100)	18(75)/0
TIMI		5(21)/2(15)	19(79)/11(85)
FRAX		1(4)/0(0)	23(96)/13 (100)
MELD		4 (17)/5(38)	20(83)/8 (62)
PERC		1(4)/0(0)	23(96)/13(100)
GWTG-Heart Failure Risk Score		5(21)/5(100)	19(79)/0(0)
*Definitions*: Question		Accurate N(%) Pre/Post	Inaccurate N(%) Pre/Post
1. When a person of color consciously or unconsciously changes their speech, behaviors, or other traits to conform to or fit in with Eurocentric society that is the definition for: (Options: Code Switching, Integration, Internalized Oppression, Internalized Racism)		13(54)/7(63)	11(46)/4(37)
2. Which of the following are example(s) of microaggression(s), check all that apply: **A female physician is mistaken for a nurse**		20(83)/12(92)	4(17)/1(8)
**“Your name is hard to pronounce, can I call you Sara?”**		22(92)/12(92)	2(8)/1(8)
**“Where are you really from?”**		23(96)/11(85)	1(4)/2(15)
3. The following statement is false: Implicit bias is an unconscious attitude that may influence behaviors		24(100)/13(100)	0(0)/0(0)
Equity is giving everyone what they need to be successful		19(79)/10(83)	5(21)/2(17)
**Race is clearly defined**		22(100)/13(100)	0/0
There is more diversity within racial groups than between racial groups		14(58)/10(100)	10(42)/0(0)
Comfort: Questions	Disagree N(%) Pre/Post	Neither N(%) Pre/Post	Agree N(%) Pre/Post
1. I know how to identify examples of institutional racism (when a program or policy gives an advantage to White people than to people of color, usually unintentionally or inadvertently).	2(9)/0	8(36)/0	14(64)/11(100)
2. I know how to identify examples of interpersonal/individual racism (i.e., using coded language, questioning a patient’s or colleague’s competence based on their race, ethnicity, or accent).	1(4)/0(0)	4(17)/0(0)	19 (79)/11(100)
3. I know how to identify examples of structural racism (i.e., homeownership as a result of centuries of structured radicalized practices, police are likely to focus on certain areas where there are predominantly black and Latinx people, etc.).	1(4)/0(0)	6(25)/1(9)	17(71)/10(91)
4. I feel comfortable talking about race.	9(38)/0	5(21)/1(11)	10(42)/8(89)
5. I am comfortable when others talk about race.	2(8)/0(0)	6(25)/1(9)	16(67)/10(91)
6. I feel like I have the resources and tools necessary to strive to achieve racial equity.	4(67)/1(17)	12(50)/2(33)	8(33)/3(50)
Departmental: Questions	Disagree N(%) Pre/Post	Neither N(%) Pre/Post	Agree N(%) Pre/Post
1. My division/department is committed to racial equity.	0(0)/0(0)	3(12)/0(0)	21(88)/11(100)
2. Our division creates an environment where everyone has equal opportunities to advance.	1(4)/0(0)	7(29)/1(9)	16(67)/10 (91)
Action: Questions		Yes N(%) Pre/Post	No N(%) Pre/Post
1. I am actively involved in advancing racial equity in my daily clinical practice and when working with medical teams.		18(75)/10(91)	6(25)/1(9)
2. I have taken the time to read, attend workshops, watch films, and educate myself about what people of color experience in this country and how I can advance racial equity in my current position.		20(83)/9(82)	4(17)/2(18)

**Table 4:** Changes in Ratings by Experience

Experience	Occasion	Beliefs	Algorithms	Definitions	Comfort	Department	Action
10+ Years Clinical Experience	Pre-curriculum	94	48	81	62	73	88
	Post-curriculum	70	59	69	83	88	75
< 10 Years Clinical Experience	Pre-curriculum	88	49	83	63	72	68
	Post-curriculum	99	64	88	76	79	89

**Table 5:** Qualitative Analysis

Theme	Quotes
***Participant awareness of race and racism in medicine***	**“***There are several studies supporting the fact that there are worse health outcomes for minorities.”* “*Sometimes patients are treated differently, sometimes they are not. It depends on the setting and situation. Not a blanket statement”*.
***Departmental racial equity needs assessment***	*“…provide(ing) additional opportunities for education on promoting equity and recognizing racism in medicine”* *“Continue (ing) to provide safe spaces for education and discussion regarding race … and how institutions and individuals can improve appreciation and understanding of cultural differences.”*
***Curriculum Effectiveness Appraisal***	“… *this curriculum is a step in the right direction*.” “*a monthly ongoing JEDI lectures*.” “It *was illuminating due to the understanding of what constitutes ‘diversity’ in the educational realm*.”
***Recommendations for Curricular change***	*“…ongoing lecture series… available on an ongoing basis.”*

**Table 6.** Strengths and Weaknesses of Repeat JEDI curriculum

Strengths	Weaknesses
Practical action plans, principles to apply	Poor attendance by department members Discussion dependent on attendance
More topics covered Relevant topics Race and research	repetitive
Good discussion in large group discussions	break out sessions
Practiced/smoothed out	

## Data Availability

The data sets generated during and/or analyzed during the current study are not publicly available to ensure the confidentiality of the participating faculty, but anonymized data are available from the corresponding author at reasonable request by a qualified investigator.

## Appendix:

### Validity Analysis:

Table 7 presents the loadings for the factor analysis solution to inform how to interpret the questionnaires. Results indicated that there may be a unidimensional trait across the questionnaires, however it does not appear all questionnaires measured the same perceptions of bias. The loadings for beliefs and definitions of bias were likely different from zero and negative (${\hat{\lambda }}_{\text{beliefs}}=-0.49$, 95% CI [−0.85, −0.11]; ${\hat{\lambda }}_{\text{definitions}}=-0.37$, 95% CI [−0.71, −0.02]), whereas all other loadings were positive. The largest loadings were for ratings of individual comfort talking about bias ($\hat{\lambda }=0.73$, 95% CI [0.39, 1.14]) and departmental commitment to equity ($\hat{\lambda }=0.81$, 95% CI [0.47, 1.15]). The loadings for awareness of the use of race in algorithms ($\hat{\lambda }=0.29$, 95% CI [−0.05, 0.82]) and personal commitment to take action to inform oneself about bias in medicine ($\hat{\lambda }=0.37$, 95% CI [−0.03, 0.77]) were positive but could not exclude the possibility of a zero relationship.

**Table 7:** Validity Analysis Results

Questionnaire	Loading ($\hat{\lambda }$)	Lower Limit	Upper Limit
Beliefs	−0.49	−0.85	−0.11
Algorithms	0.29	−0.05	0.82
Definitions	−0.37	−0.71	−0.02
Comfort	0.73	0.39	1.14
Department	0.81	0.47	1.15
Action	0.37	−0.03	0.77

*Note*: the loadings represent the extent to which the questionnaire listed in the row correlated with all other questionnaires. The upper and lower limits were from a 95% HPD credible interval. A range excluding a zero value is likely a meaningful difference.

### Measures:

Questions about beliefs about race-based discrimination and awareness of the use of race in medical algorithms were measured as ordered endorsement. Statements were provided, such as “I believe discrimination based on race occurs,” and responses were rated as “No” (scored 0), “Not sure” (scored 0.5), and “Yes” (scored 1). This allowed for an average across questions ranging from 0 (belief racism does not exist or is not used in medical algorithms) to 1 (belief racism is a problem, in general and specifically in medical algorithms). Questions about the definitions related to bias were given multiple choices and considered inaccurate (scored 0) or accurate (scored 1). An example item is “True or false: implicit bias influences behavior”. An average score of 1 indicated complete accuracy in identifying academic definitions of bias, and an average score of 0 indicated complete inaccuracy.

Questions about personal and departmental comfort were rated on a Likert scale from 1 (“Strongly disagree”) to 5 (“Strongly agree”) in response to questions like “I know how to identify institutional racism”, and “My division/department allows everybody to advance”. Raw scores were transformed into the percentage of the maximum scaling, so that a score of 1 (“Strongly disagree”) was rescored as 0, a score of 3 (“Neither agree nor disagree”) was rescored as 0.5, and a score of 5 (“Strongly agree”) was rescored as 1. This allowed for a comparison of these attitudes with other questions, all on the same proportional scale from 0 to 1.

Questions about personal action, such as “I have taken time to read or attend workshops on subjects related to racial equity”, were answered as “Yes” (scored 1) and “No” (scored 0). Other questions asked of participants were the number of years they had worked in medicine (“< 5” scored 1, “6–10” scored 2, “11–20” scored 3, and “> 20” scored 4). Lastly, participants were provided the opportunity to provide qualitative feedback.

### Evaluation:

The questionnaires were provided at two time points, before and after completing the course curriculum, with the intention that higher scores would indicate a positive evaluation of the curriculum. Aware that this is a sensitive subject, we were particularly interested in performing a validity analysis to inform how to interpret the findings appropriately. This was accomplished using multi-level modeling, modeling the average scores on the six questionnaires nested within time points nested within individuals (Harlow, 2014; Jennrich & Schluchter, 1986).

A factor analysis model was used to understand the multivariate relationships between the questionnaires. If all questionnaires showed sizable positive loadings, that would suggest that our unidimensional measurements represented awareness of bias in medicine (Harlow, 2014; Nunnally, 1978). Alternatively, if some loadings are small, this may be an indication that we only addressed some aspects of bias, such as a factual understanding of what defines a microaggression. Finally, if there are negative loadings, this would indicate that the trait of bias shows more complexity in measurement, and the implications will be considered more carefully in conjunction with the qualitative feedback.

This approach allowed us to estimate the relationships between the questionnaires jointly, and the extent to which the average scores changed across the questionnaires before and after the curriculum was implemented (Jennrich & Schluchter, 1986). While this addressed the primary research question, we also included the model experience as an ordinal measure. As there has been growing awareness of academic racism, this allowed for the opportunity to evaluate whether or not changes in understanding were equal between participants of all levels of experience.

### Estimation and Inference:

As a longitudinal design with a small class size, there were some anticipated challenges in data collection. Firstly, the class only included 34 participants, which is not large. Additionally, 25 completed the pre-curriculum survey, and 17 the post-curriculum survey, which risked a sampling bias in available information. Bayesian model estimation was used to address this significant limitation in data collection. Bayesian methodology has been emphasized for circumstances with known limitations in data collection because the final results, called the posterior distribution, are not entirely based on the observed data but a weighted average between observed data and a researcher-specified prior distribution (Hoff, 2009). Careful use of the prior can provide analytic support for data subject to the highly influential chance or biased events.

For this analysis, we used priors that emphasized the qualities of the data at pre-curriculum. Responses being more complete at that point, were suspected to be more valid indications of personal opinion, before people who may feel uncomfortable with the curriculum would stop participating. Prior means for regression intercept parameters were the means for each questionnaire pre-curriculum, and prior means for change post-curriculum and association with experience were set to zero. This reflected an assumption that the pre-curriculum averages were the only underlying true distribution, and there were no changes post-curriculum or association with years of experience. Prior variances for the regression parameters were specified to be vague, as 1,000. This was also done in an effort toward conservatism, drawing proportionally more information from the data rather than the prior. Lastly, the prior for the loadings were taken from the maximum likelihood solution of the loadings pre-curriculum, again to emphasize the more complete records for which missingness probably was less related to personal discomfort with the material.

Another way we analytically addressed bias in the data available was to estimate the model as a selection mixture model (Ibrahim & Molenberghs, 2009). This approach jointly estimates the probability that an observation will be missing to incorporate it analytically in estimating the distributions of the outcome measure. Because there were six questionnaires included in the model, we used individually implied trait scores from the factor analysis random effects to estimate the probability of missingness for each individual. Additionally, we included in the missingness model the ordinal years clinical experience and social desirability. We selected these because we thought they might be mechanisms by which participants were lost to follow up. This resulted in an analysis of and adjustment for missingness represented by individual questionnaire response style on available information, years of clinical experience, and individual personal desirability.

While these analytic approaches cannot overcome data collection limitations, they can provide needed caution by drawing results toward a solution that implies insufficient information to draw a conclusion. This helps to avoid overstating the available data. By choosing priors based on the distributions pre-curriculum this was conservative for evaluating whether or not changes post-curriculum were demonstrated, analytically weighting the results toward an assumption of no evidence of any change. Across the model, these priors emphasized the more complete data that occurred before the curriculum was implemented, which we suspected was more representative of departmental opinions. For those missing records, we modeled the probability of missingness to inform ourselves whether or not all groups were equally likely to be lost to follow up, and to address the missingness probabilistically in the final estimates.

Model estimation was performed by Gibbs sampling using SAS PROC MCMC (Ghamsary, Oda, & Beeson, 2020). A total of 300,000 samples of the posterior distribution were sampled, with 100,000 burnt in and thinning so that only every 100^th^ sample was taken, resulting in final estimates based on 2,000 samples of the posterior distribution. Trace plots were visually examined, and the effective sample size and Geweke diagnostic test were used to ensure model convergence (Hoff, 2009).

Inference was based on the 95% highest posterior density credible interval, representing the middle 95% of the data for the estimated posterior distribution. An interval that excludes a value of 0 was taken as a likely meaningful difference (Hoff, 2009). Of primary interest was the parameter for change from pre-curriculum to post-curriculum, denoted ${\hat{\beta }}_{\Delta }$, hypothesized to be positive, indicating the curriculum increased confidence and knowledge of the problems of bias in medicine. However, the interpretation of the results was primarily informed by the validity analysis of the loadings, denoted $\hat{\lambda }$, which represent whether or not there was common variation in questionnaires, providing insights into what the questionnaires represented. Lastly, the traits that were associated with missingness, denoted, $\hat{\gamma }$, demonstrate whether or not there was evidence of systematic bias in which participants were lost to follow-up.

## List of Abbreviations:

DEI: Diversity, equity and inclusion
JEDI: Justice Equity Diversity and Inclusion Curriculum
BIPOC: Black Indigenous People of Color
REDCAP: Research Electronic Data Capture
COVID-19: Coronavirus Disease 2019 (SARS-CoV-2)
IRB: Institutional Review Board
Advance-CTR: Advance Clinical and Translational Research
